# Essentiality drives the orientation bias of bacterial genes in a continuous manner

**DOI:** 10.1038/srep16431

**Published:** 2015-11-12

**Authors:** Wen-Xin Zheng, Cheng-Si Luo, Yan-Yan Deng, Feng-Biao Guo

**Affiliations:** 1 School of Biomedical Engineering, Capital Medical University, Beijing 100069, China; 2 Beijing Key Laboratory of Fundamental Research on Biomechanics in Clinical Application, Capital Medical University, Beijing 100069, China; 3 Center of Bioinformatics, School of Life Science and Technology, University of Electronic Science and Technology of China, Chengdu, 610054, China; 4 Center for Information in BioMedicine, University of Electronic Science and Technology of China, Chengdu, 610054, China; 5 Key Laboratory for Neuro Information of the Ministry of Education, University of Electronic Science and Technology of China, Chengdu, 610054, China

## Abstract

Studies had found that bacterial genes are preferentially located on the leading strands. Subsequently, the preferences of essential genes and highly expressed genes were compared by classifying all genes into four groups, which showed that the former has an exclusive influence on orientation. However, only some functional classes of essential genes have this orientation bias. Nevertheless, previous studies only performed comparative analyzes by differentiating the orientation bias extent of two types of genes. Thus, it is unclear whether the influence of essentiality on strand bias works continuously. Herein, we found a significant correlation between essentiality and orientation bias extent in 19 of 21 analyzed bacterial genomes, based on quantitative measurement of gene essentiality (or fitness). The correlation coefficient was much higher than that derived from binary essentiality measures (essential or non-essential). This suggested that genes with relatively lower essentiality, i.e., conditionally essential genes, also have some orientation bias, although it is weaker than that of absolutely essential genes. The results demonstrated the continuous influence of essentiality on orientation bias and provided details on this visible structural feature of bacterial genomes. It also proved that Geptop and IFIM could serve as useful resources of bacterial gene essentiality, particularly for quantitative analysis.

In bacterial genomes, more genes are situated on the leading strands than on the lagging strands^1^,^2^,^3^. What drives this strand bias of gene distribution has attracted much research attention in recent years. McLean *et al.* thought that gene strand-bias of bacteria was mainly caused by a preference for highly expressed genes on the leading strands^4^. Studies showed that longer operons^5^, the presence of the DNA polymerase *polC* in a genome^6^, and replication associated purine asymmetry^7^ might contribute to orientation bias. Rocha *et al.* classified genes into four categories according to expressiveness and essentiality, and found that essentiality was the primary determinant of a gene’s strand bias^8^. Lin *et al.* analyzed essential genes that were identified experimentally from 10 bacterial genomes and confirmed the previous findings that essential genes were more frequently situated on the leading strands^9^. Furthermore, the strand bias of essential genes appeared to depend on their functions. These observations proved that essentiality was the primary driving force behind gene strand bias; but these conclusions were derived from statistical tests, which lacked the correlation analysis between essentiality and strand bias. Therefore, it remains unclear whether essentiality influences the orientation bias in a continuous manner or just discretely.

Essential genes are those indispensable for an organism’s survival^10^,^11^,^12^. Systematic genome-wide interrogations, including single-gene knockouts^13^,^14^, transposon mutagenesis^15^,^16^ and RNA interference, have been used to identify essential genes^17^. A computational approach with high efficiency is an alternative to identify essential genes. Biological features associated with gene essentiality are used to predict essential genes. These features fall into three categories: intrinsic features based on sequences^18^, those derived from sequences, and data from functional omics experiments^19^,^20^,^21^,^22^. Recently, we proposed a universal method named Geptop, which applies phylogeny weighted orthology score to reflect gene essentiality and offers gene essentiality annotations^23^. This method yields good AUC scores that are higher than integrative approaches and is expected to be applied widely in all bacterial species whose genomes have been sequenced.

Usually, only binary essentiality (essential or non-essential) data are available from genome-wide experiments. Fitness of a gene provides a new perspective for quantitative analysis of gene essentiality, which may be more comprehensive than the binary essentiality. We developed a new database, Integrated quantitative Fitness Information for Microbial genes (IFIM), which currently contains data from 16 experiments and 2186 theoretical predictions^24^. In single-gene deletion mutant experiments, the contribution of a gene to fitness is usually measured as the growth rate of its deletion mutant^24^,^25^. For transposon integration libraries, the fitness of a gene was defined as the degree to which the gene tolerated transposon insertions^26^. All microbial data of transposon integrations and single-gene deletion mutants currently available were collected to derive a fitness score, which consists of the experimental entries in IFIM^24^. For most bacterial species, whose deletion/insertion mutant experimental data were not available, the result of Geptop, which was used as an alternative to genome-wide fitness data, composed the theoretical predictions in IFIM. For a certain genome, Geptop was used to predict an essentiality score (*S*) for each gene^23^. The fitness value of a gene was defined as 0 when *S* was equal to 1. When *S* was not equal to 1, the fitness was defined as 1-*S*/*S*max, where *S*max is the maximum *S* (excluding *S* = 1) in the genome^24^. The computational simulations in IFIM showed highly significant correlations with the experimentally-derived fitness data, which demonstrated that the computer-generated predictions are almost as reliable as the experimental data^24^. In this study, using the theoretical and experimental fitness in IFIM as the quantitative measure of essentiality, the correlations between gene essentiality and orientation bias were analyzed.

## Results

### Higher correlation between fitness and orientation bias than that between binary essentiality and orientation bias

Twenty-one bacterial genomes were analyzed, whose essentiality and strand bias information were available. For each gene, the fitness ranged from 0 to 1. The smaller the fitness score, the more essential the gene was. According to the annotation in the DEG database, the essentiality of a gene was denoted as 1 if the gene was essential as determined experimentally, and 0 for a non-essential gene. If the strand of a gene was 1, it meant that the gene was located on the leading strand, and 0 for a gene on the lagging strand. For each genome, we calculated the correlation coefficient of fitness-strand and binary-essentiality-stand to analyze the effect of essentiality on the orientation bias. The organisms and correlation coefficients are listed in Table 1. Nineteen chromosomes showed significant (*p* < 0.05) correlation with orientation bias and the correlation coefficients ranged from 0.175 to 0.069. The correlation coefficients of 12 chromosomes were higher than 0.1.

**Figure 1:**
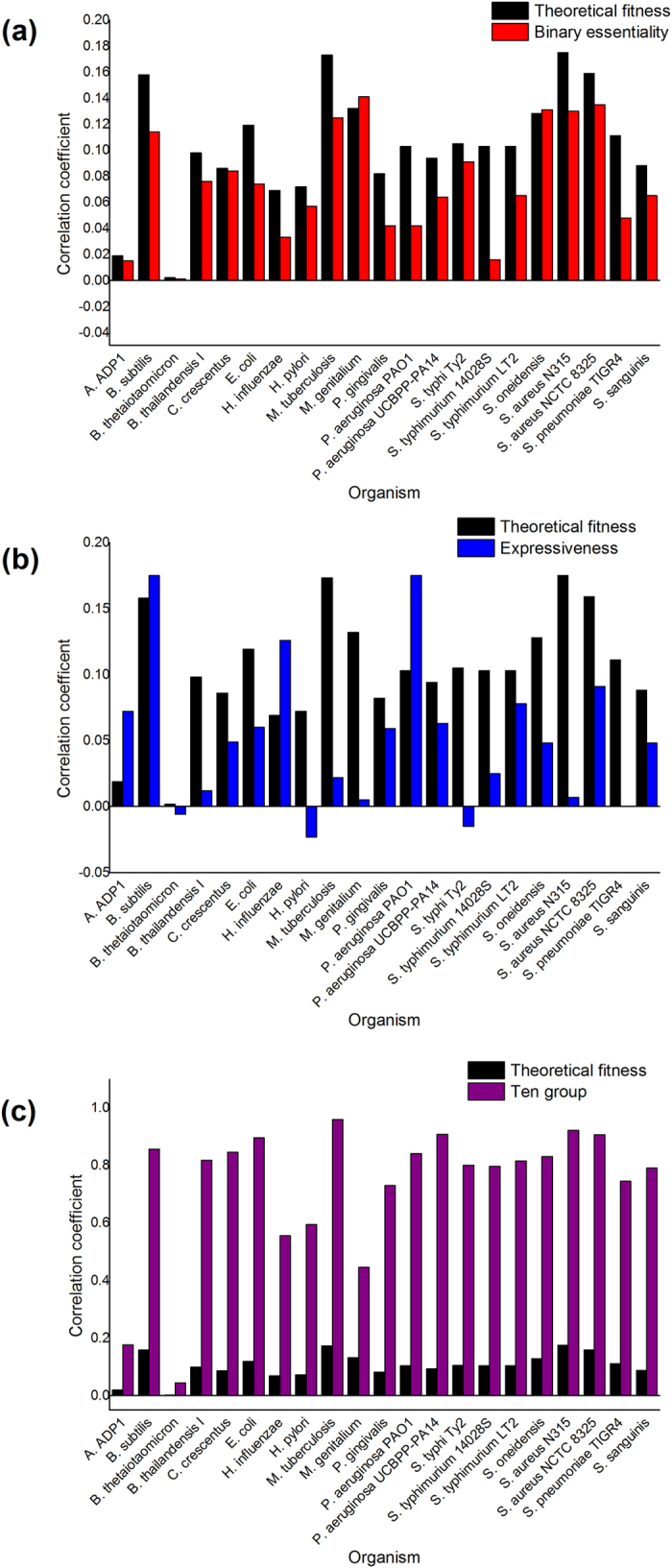
Correlation coefficients with orientation bias. **(a)** The correlation coefficients of theoretical fitness and binary essentiality with orientation bias of 21 genomes. **(b)** The correlation coefficients of theoretical fitness and expressiveness with orientation bias of 21 genomes. **(c)** The correlation coefficients of theoretical fitness before and after grouping with orientation bias of 21 genomes. The absolute value of the coefficient between fitness and strand bias was used.

According to Table 1, almost all the genomes showed a correlation with orientation bias to some degree; however, the correlation coefficients were not very high. In bacterial genomes, most genes tended to locate on the leading strands. The fitness value was inversely proportional to the essentiality. The tendency for essential genes to locate on leading strands meant that the correlation coefficients had negative values. To make the correlation coefficients of fitness agree with those of binary essentiality, we used the minus value of all the correlation coefficients between fitness and orientation bias, when the figures were plotted. The correlation coefficients between the binary essentiality and the orientation bias were plotted in Fig. 1a, together with the correlation coefficients between the theoretical fitness and the orientation bias. Compared with the binary essentiality identified experimentally, the fitness predicted theoretically showed more significant correlation with the orientation bias, and the correlation coefficients between fitness and orientation bias of 19 out of 21 chromosomes (90.5%) were higher. This demonstrated that fitness measured the essentiality more accurately than binary essentiality. The only two outliers were *Mycoplasma genitalium* G37 and *Shewanella oneidensis* MR-1. Two sample (paired) Student’s t-tests (Bilateral) indicated that the theoretical fitness showed more correlation with orientation bias than binary essentiality, with *p* = 1.72e-5. This result proved that the theoretically predicted fitness is reliable and more accurate than binary essentiality.

The correlation coefficients of experimental fitness and orientation bias are listed in Table 2. The correlation coefficients showed considerable consistency with those of theoretical predictions. Except for *Bacteroides thetaiotaomicron* VPI-5482 and *Caulobacter crescentus* NA1000, all the other correlation coefficients of theoretical fitness were slightly higher than those derived from experiments.

### Higher correlation between fitness and orientation bias than that between expressiveness and orientation bias

The correlation coefficients between expressiveness and orientation bias were also calculated for the 21 organisms (Table 1). The correlation coefficients of expressiveness and theoretical fitness were plotted in Fig. 1b, which showed that the primary influence on orientation bias is essentiality, which confirmed the conclusion of previous studies^8^,^9^. For 17 out of the 21 chromosomes (81%), fitness showed higher correlations with orientation bias than did expressiveness. By contrast, only four chromosomes showed the opposite effect: *Acinetobacter* sp. ADP1, *Bacillus subtilis* subsp*. subtilis* str. 168, *Haemophilus influenzae* Rd KW20, and *Pseudomonas aeruginosa* PAO1. The correlation coefficients between expression level and strand bias in three genomes, *B. thetaiotaomicron*, *Helicobacter pylori* 26695 and *Salmonella enterica* subsp*. enterica serovar typhi* str. Ty2, were negative, apparently indicating that highly expressed genes tended to be situated on the lagging strands. However, the P-values were all higher than 0.1, indicating that the results were not statistically significant. In addition, there were also statistically non-significant (*p* > 0.05) positive correlations in six other genomes: *Burkholderia thailandensis* E264 chromosome I, *Mycobacterium tuberculosis* H37Rv, *M. genitalium, Salmonella enterica* subsp. *enterica serovar typhimurium* str. 14028S, *Staphylococcus aureus* subsp*. aureus* N315 and *Streptococcus pneumoniae* TIGR4. Comparatively, the correlations between fitness and orientation bias were significant in 19 genomes and the correlation coefficients were consistently negative, indicating that essential genes tend to be located on leading strands in all of them. Two sample (paired) Student’s t-tests (Bilateral) indicated that fitness had more influence on orientation bias than expressiveness (*p* = 1.70e-3).

### Ten groups according to the fitness (in descending order)

Almost all the genomes showed significant correlations, but the correlation coefficients were not particularly high. This might reflect the binary denotation of the orientation bias, where 0 or 1 stands for a gene on the lagging or leading strand. If the orientation bias was represented by a continuous variable, the correlation coefficients might increase. Therefore, for each genome, we sorted all the genes according to their fitness in ascending order, and divided them into 10 groups (each group contained the same number of genes) according to their fitness scores (group 1 representing the bottom 10% and group10 representing the top 10%). For each group, the average fitness and the percentage of genes on the leading strands were calculated. Thus, the orientation bias was represented by the percentage of leading strand genes, which is a continuous variable. For each genome, the correlation coefficients between the average fitness and the percentage of leading strand genes in the 10 groups were calculated (Table 1). Consequently, almost all the genomes had much higher correlation coefficients. The correlation coefficients using data from the 10 groups were plotted in Fig. 1c, together with the correlation coefficients between the fitness and binary gene-strand bias. Obviously, the coefficients increased greatly after grouping. This confirmed the previous supposition that the binary denotation decreased the correlation coefficient, which should have a much higher value. However, the significances (P values) of the correlation coefficients of each organism did not changed significantly compared with those obtained before grouping (Table 1). Through grouping, the noise was reduced and the signal-to-noise ratio increased. Thus, the coefficients increased.

An example was constructed to prove that a binary measure representing a continuous variable would lose some information. In this example, two variables (*x* and *y*) were used. The x and y were two artificial variables without any factual meanings. They were only taken as the example to illustrate the difference of using discrete and continuous values. If we let 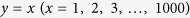, we would obtain 1000 samples 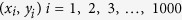. Obviously, the correlation coefficient between *x* and *y* was equal to 1. If a threshold *c*_0_ was given, the function changed to the following form:


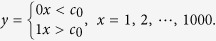


Then the correlation coefficients between 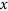 and 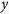 equaled 0.517, 0.692, 0.794, 0.848, 0.866, 0.849, 0.794, 0.694 and 0.522, with *p* < 0.001, which corresponded to the threshold 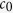 of 100, 200, 300, 400, 500, 600, 700, 800 and 900, respectively. This example showed that because of the information lost by the binary variable, the correlation coefficient decreased. Therefore, we believe that the coefficients after grouping might more reliably simulate the real case.

**Figure 2:**
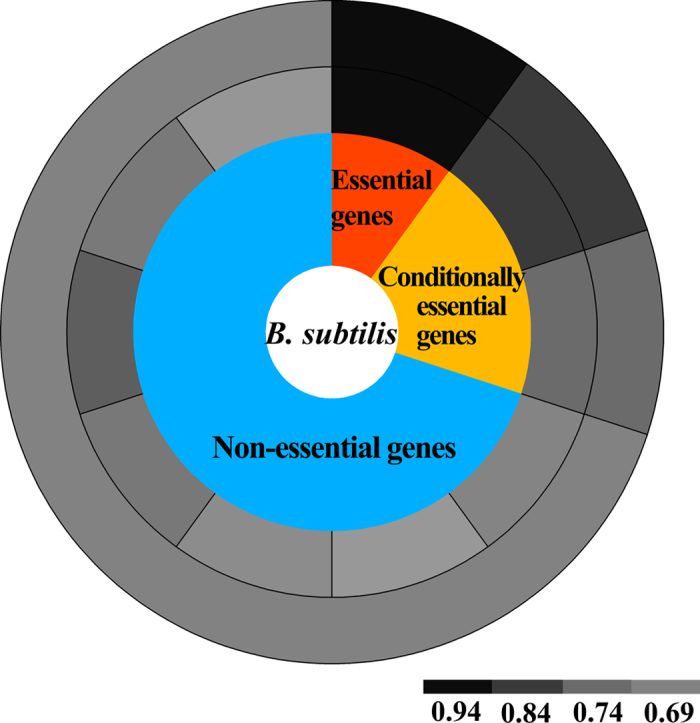
The percentage of leading strand genes of the 10 group genes for the *B. subtilis* chromosome. The 10 groups of genes were classified as essential (the first group), conditionally essential (the second and the third groups) and non-essential (the other seven groups), denoted by the inner ring. The percentage of the leading strand genes for the 10 groups and the three categories (essential, conditionally essential and non-essential) are represented by corresponding greyscale in the middle and outer rings, where the greyscale is proportional to the percentage of leading strand genes.

For each genome, the average fitness and percentage of leading strand genes of the 10 groups are listed in supplementary Table S1. For most genomes, the non-essential genes do not show leading strand bias, and the essential genes with the lowest fitness always show significant strand bias. Universally essential genes are of major importance, and are essential regardless of experimental settings. Conditionally essential genes are a set of genes that are essential under some experimental conditions, but not under other conditions^24^. Following the concept of conditionally essentiality, the genes in the groups with lowest fitness (most are between 0.1–0.3) are recognized as essential gene, whereas, the genes with a fitness value of 1 would unquestionably be regarded as non-essential genes. The genes with medium fitness values could be regarded as conditionally essential genes. Taking *B. subtilis* as an example, in supplementary Table S1, the first group of genes with an average fitness of 0.19 was the universally essential genes, which showed significant orientation bias, with 93.5% of them on leading strands. The second and third group of genes, with an average fitness of 0.84 and 0.92, were regarded as conditionally essential genes, whose percentages of leading strand genes were 84.2% and 74.1%. The other groups with higher fitness were identified as non-essential genes, showing the least orientation bias. The changing trend of leading strand percentage from absolutely essential genes, to conditionally essential genes and then to non-essential genes was strikingly illustrated in Fig. 2. In fact, supplementary Table S1 shows that for most chromosomes, the orientation bias is significant in essential genes, less significant in conditionally essential genes, but still more than for the non-essential genes. The non-essential gene group shows the least orientation bias. This result confirms the previous supposition that a continuous measure of essentiality (fitness) is more accurate than binary essentiality, and the influence on orientation bias is also continuous.

In addition, we also grouped genes in ten equal intervals of their fitness values. The average fitness and percentage of leading strand genes of the 10 groups were calculated for each genome. The total number of genes and the number of genes on leading strand of each group were also listed in supplementary Table S2. The correlation coefficients between the average fitness and orientation bias after grouping in two ways (to have equal number of genes in each group or to group according to their fitness range) were calculated and listed in supplementary Table S3. In general the results of the two gene grouping ways agree with each other, except for four organisms (*Escherichia coli* str. K-12 substr. MG1655, *M. genitalium*, *Staphylococcus aureus* subsp. aureus NCTC 8325, *Streptococcus sanguinis* SK36). Take *M. genitalium* as an example, the third, forth, fifth and sixth groups contain only 1, 2, 4 and 3 genes, with a percentage of leading strand genes of 0, 1, 0.75 and 0.333, which weaken the linear correlation.

## Discussion

The genes in a bacterial genome can be divided into two groups, essential genes and non-essential genes, based on genome-wide experiments. In fact, some genes are dispensable only in certain environments, and are termed conditionally essential gene^24^. Obviously, these genes are not as essential as the absolutely essential genes, but are more important than the non-essential genes, which suggests that a quantitative measure is needed to describe the importance of a gene. Quantitative essentiality carries more information than binary essentiality, and is more accurate.

Geptop can predict essential genes based on their phylogeny weighting orthology scores^23^. Geptop yields good AUC scores that are higher than integrative approaches and could be applied widely in most bacterial species whose genomes have been completely sequenced. The essential genes predicted by the Geptop have many features, such as higher codon bias, higher distribution bias and abundant protein–protein interactions, indicating its reliability^23^. IFIM, which allows easy access to fitness data of microbial genes, contains data from 16 experiments and 2186 theoretical predictions^24^. The highly significant correlation between the experimentally-derived fitness and the computational simulations demonstrated that the computer-generated predictions were often as reliable as the experimental data^24^, meaning that fitness can be used as a more complete description of the essentiality of a microbial gene than binary essentiality. In this study, the correlation between the essentiality and the gene-strand bias was analyzed by calculating the correlation coefficient, which is more accurate than comparing the percentage of several groups of genes on leading strands. Table 1 and 2 show that the correlation coefficients of theoretical fitness and experimental fitness agree with each other. In most cases, the correlation coefficients of the theoretical fitness are slightly higher than those of the experimental fitness. This result confirms that the fitness predicted theoretically is as reliable as the experimentally-derived fitness, which can describe the essentiality of a gene more accurately, especially when the experimentally-derived fitness is unavailable.

Although previous studies revealed a determinant influence of essentiality on orientation bias in bacteria, they did not demonstrate whether it acts in a continuous manner or just discretely (logically)^8^. Fitness is a quantitative measurement describing essentiality in a continuous way, rather than a logical value (essential or non-essential). With this measure, it was feasible to test whether the continuous effect exists or not. If essentiality indeed affects gene-strand bias in a continuous manner, the correlation between fitness and gene-strand bias should be higher than that of binary essentiality. The results in Table 1 and Fig. 1 validated our hypothesis. The average fitness and the percentage of the leading strand genes are listed in supplementary Table S1. The gene in the first group with the lowest average fitness is always an essential gene. Consequently, the essential gene groups in almost all genomes show an obvious gene-strand bias. The genes in the last several groups (with fitness values of 1) always show lowest leading strand percentages and their percentages are similar. The genes in the middle groups, with an average fitness ranging between the lowest average fitness and 1, could be recognized as conditionally essential genes. Some of the conditionally essential genes prefer leading strands. Thus, essentiality shows a continuous influence on gene-strand bias. Fitness, the quantitative measurement predicted theoretically, is more reliable and carries more information than logical essentiality.

Fig. 1b shows that for most chromosomes, fitness has a higher correlation coefficient with gene-strand bias, compared with expressiveness. Correlation after grouping illustrated that the essentiality might explain over 50% of the whole orientation bias in 16 of the 21 analyzed bacteria, which could be quantitatively estimated from the values of *R*^*2*^. Therefore, essentiality plays a key role in shaping the gene strand bias in most bacterial genomes, rather than expressiveness, which agreed with the conclusion of Rocha *et al.*^8^. Genes tend to be located on the leading strands rather than the lagging strands in bacterial genomes^8^. Mao *et al.* suggest that (i) genes of some functional categories, such as ribosomal gene, have a greater tendency to be on the leading strand; (ii) by contrast, other categories, such as transcription factors tend to be on the lagging strands; (iii) there is a balancing force that prevents all genes from moving to the leading strand; and (iv) the percentage of leading strand genes can be accurately explained based on the numbers of genes in the functional categories listed in (i) and (ii), genome size and gene density^27^. If an artificial functional category was created, this category would have a significant P value for a tendency to be on the leading strand^27^. Lin *et al.* suggest that the functionalities likely play a key role in orientation bias^9^. Chen *et al.* suggest that the existence of lagging strand encoded genes could be explained by a balance between deleterious mutations that bring genes from the leading to the lagging strands, and purifying selection purging such mutants^28^,^29^. Our results confirmed that essentiality was a dominant factor for orientation bias in most (16 of 21) bacteria, with *R*^*2*^ > 0.5 after grouping correlation analysis. However, as Mao *et al.* suggested, the balance of multiple factors may play roles in a few (5 of 21) genomes, where *R*^*2*^ is much lower than 0.5, even in the correlation analyzes after grouping.

## Methods

DEG is a database that contains all available essential genes that have been determined experimentally at the genome scale^17^,^30^. The bacterial annotation information of 21 organisms was downloaded from NCBI ftp site (ftp://ftp.ncbi.nih.gov/genomes/Bacteria/), from which the location and the strand (Watson or Crick) information could be obtained. Compared with the essential genes recorded in the DEG database for each genome, all the genes that had not been recorded as essential genes were regarded as non-essential genes. Thus, the binary essentiality information for each organism was obtained.

The IFIM database currently contains fitness data from 16 experiments and 2186 theoretical predictions, which can be used as a quantitative measurement of essentiality. The computer-generated predictions show highly significant correlations with the experimentally-derived fitness data; therefore, they can be used as a reliable alternative when experimental data are unavailable.

The IDs of the 21 bacterial genomes in IFIM are the same with the accession numbers in NCBI. The NCBI accession number and the GEO (Expression level database at NCBI) number of the chromosomes used in this study were listed in Table 1 31. In fact, there are 36 genomes with genome scale essentiality data in the DEG database. However, we take comparison of the effect of essentiality and expression level on strand bias as one of the emphases and there are some bacteria without genome-wide microarray data. Finally, we only consider the 21 genomes that have expressiveness data (microarray data) in GEO.

To determine the DNA sequences of the leading strand and the lagging strand for each bacterial genome, the replication origin and replication termini were needed, which could be obtained from the DoriC database^32^,^33^. According to the annotation from NCBI, for a gene on the Watson strand, if the gene was located in the region from the replication origin to the replication termini, the gene was on the leading strand, and if the gene was located in the region from the termini to the origin, the gene was on the lagging strand. For a gene on the Crick strand, a gene located in the region from the origin to the termini was on the lagging strand, and a gene located in the region from the termini to the origin was on the leading strand.

Twenty-one bacteria were completely annotated in all the databases. For each gene of the 21 bacterial genomes, the fitness was gained from the IFIM database^24^, and the binary essentiality from experiments was determined using the data in the DEG database^17^. The expression level data were extracted from the NCBI GEO database^31^. The genes of the leading strand or the lagging strand were determined using the location information in NCBI together with the replication position information in the DoriC database^32^. The fitness, binary essentiality, expressiveness and the strand (leading or lagging) for each gene of the 21 analyzed organisms were listed in supplementary Table S4.

The relationships between the essentiality (quantitative measurement fitness and binary essentiality) and orientation bias were analyzed by calculating the Pearson correlation coefficients, together with their significances, using the R software (http://www.r-project.org/).

## Conclusion

In this study, for the first time, correlation analyzes were performed between essentiality and gene orientation bias in bacteria. The correlations between fitness and gene orientation bias are significantly higher than that between binary essentiality and gene orientation bias, which was confirmed by Two sample (paired) Student’s t-tests (Bilateral; *p* = 1.72e–5). This result suggested that essentiality acts continuously on gene orientation. After classifying all genes into 10 groups according to their fitness values, each group was assigned a quantitative value rather than a logical value of strand preference. Higher correlations were achieved in correlation analyzes after grouping. This result suggested that essentiality explained over 50% of gene orientation bias in most bacterial genomes. However, multiple balancing factors might operate in a few bacteria. We believe that this work provides supplementary details on the influence of essentiality on gene orientation bias in bacteria.

## Additional Information

**How to cite this article**: Zheng, W.-X. *et al.* Essentiality drives the orientation bias of bacterial genes in a continuous manner. *Sci. Rep.* **5**, 16431; doi: 10.1038/srep16431 (2015).

## Supplementary Material

Supplementary Table S1 S2 S3

Supplementary Table S4

## Figures and Tables

**Table 1:** Information of the 21 organisms used in this analysis and their correlation coefficients with orientation bias.

Organism				Essentiality (binary)		Fitness (theoretical)1 group		Fitness (theoretical)10 groups		Expressiveness	
No	Name	Accession number	GEO number	>R	P value	R	P value	R	P value	R	P value
1	*Acinetobacter* ADP1	NC_005966	GPL16388	0.015	3.75e-1	−0.019	2.75e-1	−0.177	6.24e-1	0.072	3.11e-5
2	*Bacillus subtilis* 168	NC_000964	GPL6257	0.114	1.67e-13	−0.158	1.02e-24	−0.857	1.52e-3	0.175	4.98e-28
3	*Bacteroides thetaiotaomicron* VPI-5482	NC_004663	SRX375058	0.001	9.39e-1	−0.002	8.81e-1	−0.045	9.01e-1	−0.006	6.83e-1
4	*Burkholderia thailandensis* E264 chromosome I	NC_007651	GPL18529	0.076	1.46e-5	−0.098	1.77e-8	−0.818	3.85e-3	0.012	5.09e-1
5	*Caulobacter crescentus* NA1000	NC_011916	SRX534373 (GSM1381200)	0.084	2.49e-7	−0.086	1.12e-7	−0.846	2.06e-3	0.049	2.61e-3
6	*Escherichia coli* K-12 MG1655	NC_000913	SRX996440	0.074	1.71e-6	−0.119	1.40e-14	−0.896	4.43e-4	0.060	4.93e-3
7	*Haemophilus influenzae* Rd KW20	NC_000907	SRX026485	0.033	1.87e-1	−0.069	5.62e-3	−0.556	9.49e-2	0.126	4.36e-7
8	*Helicobacter pylori* 26695	NC_000915	GPL19991	0.057	02.96e-2	−0.072	5.72e-3	−0.595	6.94e-2	−0.023	3.85e-1
9	*Mycobacterium tuberculosis* H37Rv	NC_000962	SRX902301 (GSM1625709)	0.125	4.44e-15	−0.173	2.30e-27	−0.960	1.02e-5	0.022	1.86e-1
10	*Mycoplasma genitalium* G37	NC_000908	ERX452667	0.141	2.08e-3	−0.132	4.05e-3	−0.446	1.96e-1	0.005	9.16e-1
11	*Porphyromonas gingivalis* ATCC 33277	NC_010729	GPL18306	0.042	5.69e-2	−0.082	1.80e-4	−0.730	1.65e-2	0.059	2.07e-2
12	*Pseudomonas aeruginosa* PAO1	NC_002516	SRX597269 (GSM1412555)	0.042	1.85e-3	−0.103	2.18e-14	−0.841	2.31e-3	0.175	4.98e-39
13	*Pseudomonas aeruginosa* UCBPP-PA14	NC_008463	SRX352172 (GSM1232747)	0.064	7.97e-7	−0.094	5.79e-13	−0.908	2.79e-4	0.063	4.91e-6
14	*Salmonella typhi* Ty2	NC_004631	ERP006542	0.091	1.49e-9	−0.105	4.22e-12	−0.800	5.48e-3	−0.015	3.36e-1
15	*Salmonella typhimurium* 14028S	NC_016856	GPL20046	0.016	2.44e-1	−0.103	4.04e-14	−0.797	5.82e-3	0.025	6.65e-2
16	*Salmonella typhimurium* LT2	NC_003197	GSE45445	0.065	1.64e-5	−0.103	6.86e-12	−0.816	4.01e-3	0.078	3.15e-7
17	*Shewanella oneidensis* MR-1	NC_004347	GSE25821	0.131	1.27e-14	−0.128	4.49e-14	−0.831	2.87e-3	0.048	5.15e-3
18	*Staphylococcus aureus* N315	NC_002745	SRX803166 (GSM1563062)	0.130	3.11e-11	−0.175	2.76e-19	−0.921	1.57e-4	0.007	7.13e-1
19	*Staphylococcus aureus* NCTC 8325	NC_007795	SRX529241 (GSM1376717)	0.135	9.05e-13	−0.159	4.77e-17	−0.907	2.87e-4	0.091	2.10e-6
20	*Streptococcus pneumoniae* TIGR4	NC_003028	SRX109959	0.048	2.91e-2	−0.111	3.10e-7	−0.745	1.35e-2	0.0003	9.88e-1
21	*Streptococcus sanguinis* SK36	NC_009009	GSE25340	0.065	1.95e-3	−0.088	2.59e-5	−0.791	6.38e-3	0.048	2.29e-2

**Table 2:** Correlation coefficients of the experimental fitness and orientation bias.

Organism		Experimental fitness		
No.	Abbr.	Experiment	*R*	P value
1	*A.* ADP1	AB01	−0.016	4.07e-1
3	*B. thetaiotaomicron*	BT01	0.027	1.99e-1
5	*C. crescentus*	CC01	−0.111	6.89e-12
6	*E. coli*	EC01	−0.095	1.86e-9
		EC02	−0.093	3.05e-9
		EC03	−0.055	4.89e-4
8	*H. pylori*	HP01	−0.021	4.28e-1
13	*P. aeruginosa* UCBPP-PA14	PA01	−0.052	3.27e-4
14	*S. typhi* Ty2	STY01	−0.069	5.81e-6
		STY02	-0.075	8.76e-7
		STY03	−0.077	4.40e-7
15	*S. typhimurium* 14028S	STM01	−0.057	3.71e-5
