# Supplementary material for: Essentiality drives the orientation bias of bacterial genes in a continuous manner
Source: Sci Rep. 2015 Nov 12;5:16431. doi: 10.1038/srep16431 (PMC4642330; doi:10.1038/srep16431)
Supplement: Supplementary Table S1 S2 S3 [file srep16431-s1.pdf]

Essentiality drives the orientation bias of bacterial genes in a continuous manner

Wen-Xin Zheng[1,2], Cheng-Si Luo[3,4,5,6], Yan-Yan Deng[3,4,5,6], and Feng-Biao Guo[3,4,5,6]*

[1] School of Biomedical Engineering, Capital Medical University, Beijing 100069, China
[2] Beijing Key Laboratory of Fundamental Research on Biomechanics in Clinical Application, Capital Medical University, Beijing 100069, China
[3] Center of Bioinformatics, School of Life Science and Technology, University of Electronic Science and Technology of China, Chengdu, 610054, China
[4] Health Big Data Science Research Center, Big Data Research Center, University of Electronic Science and Technology of China, Chengdu, 610054, China
[5] Center for Information in BioMedicine, University of Electronic Science and Technology of China, Chengdu, 610054, China
[6] Key Laboratory for Neuro Information of the Ministry of Education, University of Electronic Science and Technology of China, Chengdu, 610054, China

Corresponding author.

E-mail: fbguo@uestc.edu.cn; Tel: 86-28-83202351; Fax: 86-28-83202351.

Supplementary Table S1. Average fitness and percentage of the leading strand gene of 10 groups in ascending order of average fitness for the 21 organisms.

A. ADP1	Percentage	0.606	0.633	0.588	0.619	0.619	0.624	0.624	0.636	0.564	0.563
	Fitness	0	0.297	0.875	0.911	0.985	1	1	1	1	1
B. subtilis	Percentage	0.935	0.842	0.741	0.691	0.650	0.674	0.715	0.767	0.712	0.655
	Fitness	0.189	0.843	0.915	0.977	1	1	1	1	1	1
B. thetaiotaomicron	Percentage	0.583	0.570	0.597	0.566	0.642	0.595	0.549	0.566	0.589	0.538
	Fitness	0.502	0.900	0.934	1	1	1	1	1	1	1
B. thailandensis I	Percentage	0.697	0.627	0.544	0.596	0.587	0.563	0.550	0.581	0.627	0.554
	Fitness	0.344	0.812	0.922	0.966	1	1	1	1	1	1
C. crescentus	Percentage	0.660	0.580	0.553	0.484	0.487	0.537	0.553	0.564	0.519	0.534
	Fitness	0.257	0.736	0.936	0.969	1	1	1	1	1	1
E. coli	Percentage	0.712	0.591	0.528	0.511	0.513	0.567	0.571	0.506	0.496	0.496
	Fitness	0.303	0.871	0.942	0.957	1	1	1	1	1	1
H. influenzae	Percentage	0.694	0.525	0.519	0.575	0.575	0.525	0.556	0.5	0.569	0.470
	Fitness	0.083	0.260	0.307	0.325	0.652	0.895	0.949	0.987	1	1
H. pylori	Percentage	0.705	0.582	0.589	0.548	0.5	0.630	0.616	0.562	0.548	0.5
	Fitness	0.136	0.300	0.487	0.802	0.921	0.95	0.994	1	1	1
M. tuberculosis	Percentage	0.827	0.642	0.567	0.590	0.546	0.557	0.544	0.534	0.570	0.529
	Fitness	0.289	0.592	0.922	0.972	1	1	1	1	1	1
M. genitalium	Percentage	0.702	0.957	0.915	0.936	0.787	0.872	0.723	0.830	0.574	0.804
	Fitness	0	0	0	0	0	0	0	0	0.698	0.963
P. gingivalis	Percentage	0.639	0.495	0.538	0.481	0.471	0.476	0.438	0.534	0.519	0.546
	Fitness	0.226	0.701	0.876	0.922	0.952	1	1	1	1	1
P. aeruginosa PAO1	Percentage	0.682	0.575	0.530	0.574	0.550	0.535	0.555	0.581	0.485	0.528
	Fitness	0.480	0.883	0.938	1	1	1	1	1	1	1
P. aeruginosa UCBPP-PA14	Percentage	0.686	0.555	0.528	0.509	0.523	0.538	0.497	0.565	0.477	0.533
	Fitness	0.219	0.883	0.942	1	1	1	1	1	1	1
S. typhi Ty2	Percentage	0.724	0.598	0.554	0.536	0.639	0.510	0.566	0.602	0.547	0.535
	Fitness	0.334	0.849	0.938	0.959	1	1	1	1	1	1
S. typhimurium 14028S	Percentage	0.716	0.571	0.542	0.573	0.567	0.492	0.618	0.582	0.561	0.508
	Fitness	0.465	0.878	0.937	0.995	1	1	1	1	1	1
S. typhimurium LT2	Percentage	0.716	0.604	0.547	0.556	0.570	0.554	0.561	0.633	0.599	0.517
	Fitness	0.396	0.833	0.941	0.964	1	1	1	1	1	1
S. oneidensis	Percentage	0.730	0.551	0.568	0.519	0.603	0.574	0.539	0.519	0.504	0.467
	Fitness	0.364	0.813	0.921	0.941	1	1	1	1	1	1
S. aureus N315	Percentage	0.926	0.864	0.771	0.767	0.643	0.698	0.686	0.733	0.717	0.674
	Fitness	0	0.411	0.828	0.925	0.956	1	1	1	1	1
S. aureus NCTC 8325	Percentage	0.942	0.836	0.764	0.764	0.742	0.695	0.738	0.793	0.731	0.674
	Fitness	0.126	0.644	0.909	0.951	0.986	1	1	1	1	1
S. pneumoniae	Percentage	0.910	0.857	0.890	0.805	0.781	0.705	0.805	0.762	0.790	0.723
	Fitness	0.071	0.578	0.848	0.928	0.934	1	1	1	1	1
S. sanguinis	Percentage	0.907	0.903	0.858	0.827	0.796	0.783	0.814	0.850	0.805	0.791
	Fitness	0.002	0.615	0.858	0.916	0.930	1	1	1	1	1

Supplementary Table S2. Average fitness, percentage of the leading strand genes, total number of genes in one group and the number of leading strand genes of 10 groups for each genome. The genes were grouped according to the fitness ranges in ascending order.

Fitness range		[0,0.1)	[0.1,0.2)	[0.2,0.3)	[0.3,0.4)	[0.4,0.5)	[0.5,0.6)	[0.6,0.7)	[0.7,0.8)	[0.8,0.9)	[0.9,1.0]
A. ADP1	Percentage	0.629	1.000	0.333	0.923	0.750	0.600	0.667	0.500	0.594	0.605
	Fitness	0.000	0.114	0.247	0.347	0.436	0.553	0.661	0.741	0.824	0.979
	Total number	504	2	6	13	4	30	24	70	138	2515
	Number of leading strand genes	317	2	2	12	3	18	16	35	82	1521
B. subtilis	Percentage	0.930	1.000	1.000	1.000	1.000	1.000	0.885	0.946	0.821	0.705
	Fitness	0.000	0.158	0.262	0.379	0.449	0.532	0.646	0.753	0.833	0.984
	Total number	270	4	14	17	16	23	61	92	207	3466
	Number of leading strand genes	251	4	14	17	16	23	54	87	170	2442
B. thetaiotaomicron	Percentage	0.784	0.473	0.500	0.551	0.556	0.553	0.645	0.561	0.592	0.579
	Fitness	0.061	0.157	0.257	0.368	0.452	0.538	0.639	0.751	0.848	0.986
	Total number	51	55	38	49	18	47	31	132	201	4156
	Number of leading strand genes	40	26	19	27	10	26	20	74	119	2408
B. thailandensis I	Percentage	0.885	0.860	0.658	0.679	0.783	0.541	0.450	0.652	0.632	0.574
	Fitness	0.061	0.156	0.252	0.362	0.450	0.549	0.638	0.752	0.847	0.986
	Total number	52	57	38	53	23	61	40	135	182	2634
	Number of leading strand genes	46	49	25	36	18	33	18	88	115	1513
C. crescentus	Percentage	0.726	0.672	0.683	0.519	0.691	0.635	0.591	0.600	0.557	0.528
	Fitness	0.053	0.136	0.235	0.348	0.455	0.543	0.647	0.749	0.863	0.989
	Total number	84	64	60	77	68	148	22	30	221	2992
	Number of leading strand genes	61	43	41	40	47	94	13	18	123	1580
E. coli	Percentage	0.850	0.710	0.569	0.684	0.536	0.783	0.794	0.676	0.589	0.523
	Fitness	0.056	0.148	0.248	0.347	0.435	0.554	0.652	0.756	0.874	0.987
	Total number	113	62	72	38	28	23	34	71	353	3342
	Number of leading strand genes	96	44	41	26	15	18	27	48	208	1748
H. influenzae	Percentage	0.796	0.542	0.531	0.526	0.644	0.667	0.633	0.595	0.515	0.522
	Fitness	0.043	0.153	0.272	0.317	0.454	0.556	0.649	0.741	0.856	0.975
	Total number	98	72	209	249	45	27	30	37	99	738
	Number of leading strand genes	78	39	111	131	29	18	19	22	51	385
H. pylori	Percentage	0.726	0.722	0.574	0.603	0.712	0.563	0.512	0.500	0.623	0.555
	Fitness	0.044	0.156	0.262	0.317	0.442	0.540	0.648	0.750	0.842	0.976
	Total number	62	36	101	116	52	48	43	36	77	889
	Number of leading strand genes	45	26	58	70	37	27	22	18	48	493
M. tuberculosis	Percentage	0.931	0.937	0.875	0.800	0.747	0.620	0.727	0.667	0.594	0.553
	Fitness	0.046	0.145	0.248	0.357	0.458	0.520	0.653	0.754	0.865	0.991
	Total number	72	63	40	75	150	263	22	36	180	2984
	Number of leading strand genes	67	59	35	60	112	163	16	24	107	1651
M. genitalium	Percentage	0.839	0.810	0.000	1.000	0.750	0.333	0.667	0.700	0.550	0.774
	Fitness	0.000	0.100	0.252	0.338	0.458	0.555	0.693	0.765	0.841	0.962
	Total number	378	0*	1	2	4	3	3	10	20	53
	Number of leading strand genes	317	0	0	2	3	1	2	7	11	41
P. gingivalis	Percentage	0.765	0.554	0.675	0.676	0.476	0.489	0.548	0.488	0.563	0.494
	Fitness	0.060	0.157	0.257	0.374	0.452	0.540	0.639	0.755	0.848	0.979
	Total number	51	56	40	37	21	45	31	121	144	1542
	Number of leading strand genes	39	31	27	25	10	22	17	59	81	762

P. aeruginosa PAO1	Percentage	0.861	0.847	0.717	0.848	0.654	0.638	0.613	0.594	0.561	0.544
	Fitness	0.047	0.144	0.248	0.339	0.431	0.537	0.653	0.762	0.858	0.989
	Total number	36	59	60	46	52	58	93	217	228	4665
	Number of leading strand genes	31	50	43	39	34	37	57	129	128	2537
P. aeruginosa UCBPP-PA14	Percentage	0.684	0.815	0.818	0.682	0.700	0.700	0.500	0.640	0.536	0.524
	Fitness	0.003	0.157	0.253	0.353	0.451	0.546	0.637	0.754	0.847	0.988
	Total number	351	27	22	22	10	40	28	139	209	5044
	Number of leading strand genes	240	22	18	15	7	28	14	89	112	2644
S. typhi Ty2	Percentage	0.892	0.781	0.672	0.667	0.655	0.600	0.750	0.625	0.601	0.561
	Fitness	0.062	0.153	0.254	0.360	0.442	0.551	0.656	0.759	0.860	0.987
	Total number	74	73	67	54	58	55	28	80	391	3472
	Number of leading strand genes	66	57	45	36	38	33	21	50	235	1948
S. typhimurium 14028S	Percentage	0.923	0.860	0.739	0.649	0.685	0.676	0.678	0.652	0.581	0.555
	Fitness	0.051	0.154	0.247	0.349	0.446	0.548	0.639	0.720	0.858	0.989
	Total number	39	50	69	37	54	68	87	155	327	4428
	Number of leading strand genes	36	43	51	24	37	46	59	101	190	2457
S. typhimurium LT2	Percentage	0.917	0.800	0.800	0.661	0.705	0.651	0.701	0.640	0.579	0.567
	Fitness	0.048	0.149	0.252	0.347	0.459	0.544	0.648	0.753	0.871	0.988
	Total number	48	30	55	56	61	166	67	75	328	3563
	Number of leading strand genes	44	24	44	37	43	108	47	48	190	2020
S. oneidensis	Percentage	0.868	0.855	0.730	0.680	0.667	0.712	0.591	0.574	0.534	0.537
	Fitness	0.062	0.156	0.254	0.361	0.445	0.548	0.640	0.756	0.847	0.983
	Total number	53	55	37	50	24	66	44	155	206	2766
	Number of leading strand genes	46	47	27	34	16	47	26	89	110	1484
S. aureus N315	Percentage	0.906	0.955	0.933	0.950	1.000	0.909	0.835	0.782	0.752	0.704
	Fitness	0.001	0.158	0.239	0.351	0.453	0.546	0.643	0.757	0.852	0.982
	Total number	308	22	15	20	19	33	79	110	145	1829
	Number of leading strand genes	279	21	14	19	19	30	66	86	109	1287
S. aureus NCTC 8325	Percentage	0.977	0.949	0.864	0.833	0.500	0.938	0.933	0.857	0.786	0.735
	Fitness	0.041	0.142	0.266	0.336	0.467	0.571	0.660	0.761	0.840	0.984
	Total number	129	78	66	72	2	16	30	91	117	2156
	Number of leading strand genes	126	74	57	60	1	15	28	78	92	1585
S. pneumoniae	Percentage	0.882	0.953	0.891	0.833	0.885	0.905	0.805	0.860	0.897	0.769
	Fitness	0.006	0.150	0.266	0.363	0.461	0.557	0.654	0.773	0.850	0.980
	Total number	127	64	46	24	26	21	41	93	175	1486
	Number of leading strand genes	112	61	41	20	23	19	33	80	157	1142
S. sanguinis	Percentage	0.910	0.944	0.846	0.818	0.889	0.895	0.900	0.930	0.865	0.810
	Fitness	0.000	0.148	0.256	0.350	0.445	0.552	0.649	0.753	0.838	0.976
	Total number	222	18	13	11	18	19	40	86	200	1641
	Number of leading strand genes	202	17	11	9	16	17	36	80	173	1329

* If a group doesn't contains any gene, the average fitness of this group is represented by the minimum value of this group and the percentage of leading strand genes is represented by the average percentage of the leading strand genes of the whole genome.

Supplementary Table S3. Correlation coefficients between fitness and orientation bias after grouping in a way to have equal number of genes in each group and in a way that genes in a particular group have similar fitness values.

Organism		Fitness (theoretical)			
		10 groups (equal number)		10 groups (fitness range)	
No.	Name	*R*	P value	*R*	P value
1	*Acinetobacter* ADP1	-0.177	6.24e-1	-0.303	3.95e-1
2	*Bacillus subtilis* 168	-0.857	1.52e-3	-0.709	2.16e-2
3	*Bacteroides thetaiotaomicron* VPI-5482	-0.045	9.01e-1	-0.091	8.03e-1
4	*Burkholderia thailandensis* E264 chromosome I	-0.818	3.85e-3	-0.699	2.44e-2
5	*Caulobacter crescentus* NA1000	-0.846	2.06e-3	-0.724	1.80e-2
6	*Escherichia coli* K-12 MG1655	-0.896	4.43e-4	-0.424	2.22e-1
7	*Haemophilus influenzae* Rd KW20	-0.556	9.49e-2	-0.410	2.40e-1
8	*Helicobacter pylori* 26695	-0.595	6.94e-2	-0.653	4.06e-2
9	*Mycobacterium tuberculosis* H37Rv	-0.960	1.02e-5	-0.942	4.69e-5
10	*Mycoplasma genitalium* G37	-0.446	1.96e-1	-0.044	9.05e-1
11	*Porphyromonas gingivalis* ATCC 33277	-0.730	1.65e-2	-0.668	3.48e-2
12	*Pseudomonas aeruginosa* PAO1	-0.841	2.31e-3	-0.918	1.82e-4
13	*Pseudomonas aeruginosa* UCBPP-PA14	-0.908	2.79e-4	-0.766	9.75e-3
14	*Salmonella typhi* Ty2	-0.800	5.48e-3	-0.776	8.34e-3
15	*Salmonella typhimurium* 14028S	-0.797	5.82e-3	-0.900	3.85e-4
16	*Salmonella typhimurium* LT2	-0.816	4.01e-3	-0.912	2.41e-4
17	*Shewanella oneidensis* MR-1	-0.831	2.87e-3	-0.946	3.56e-5
18	*Staphylococcus aureus* N315	-0.921	1.57e-4	-0.813	4.25e-3
19	*Staphylococcus aureus* NCTC 8325	-0.907	2.87e-4	-0.348	3.25e-1
20	*Streptococcus pneumoniae* TIGR4	-0.745	1.35e-2	-0.563	9.00e-2
21	*Streptococcus sanguinis* SK36	-0.791	6.38e-3	-0.333	3.46e-1
